# Targeting androgen receptor (AR) with antiandrogen Enzalutamide increases prostate cancer cell invasion yet decreases bladder cancer cell invasion via differentially altering the AR/circRNA-ARC1/miR-125b-2-3p or miR-4736/PPARγ/MMP-9 signals

**DOI:** 10.1038/s41418-021-00743-w

**Published:** 2021-06-14

**Authors:** Gang Deng, Ronghao Wang, Yin Sun, Chi-Ping Huang, Shuyuan Yeh, Bosen You, Changyong Feng, Gonghui Li, Shenglin Ma, Chawnshang Chang

**Affiliations:** 1 Department of Urology, Affiliated Hangzhou First People’s Hospital, Zhejiang University School of Medicine, Hangzhou, China; 2 George Whipple Lab for Cancer Research, Departments of Pathology, Urology, Radiation Oncology and The Wilmot Cancer Institute, University of Rochester Medical Center, Rochester, NY USA; 3 Department of Biochemistry and Molecular Biology, School of Basic Medical Sciences, Southwest Medical University, Luzhou, China; 4 Department of Urology, China Medical University/Hospital, Taichung, Taiwan; 5 Department of Biostatistics, University of Rochester Medical Center, Rochester, NY USA

**Keywords:** Cancer models, Metastasis

## Abstract

Androgen-deprivation therapy (ADT) via targeting androgens/androgen receptor (AR) signals may suppress cell proliferation in both prostate cancer (PCa) and bladder cancer (BCa), yet its impact on the cell invasion of these two urological cancers remains unclear. Here we found targeting androgens/AR with either the recently developed antiandrogen Enzalutamide (Enz) or AR-shRNAs led to increase PCa cell invasion, yet decrease BCa cell invasion. Mechanistic dissection revealed that suppressing androgens/AR signals could result in differential alterations of the selective circular RNAs (circRNAs) as a result of differential endogenous AR transcription. A negative autoregulation in PCa, yet a positive autoregulation in BCa, as a result of differential binding of AR to different androgen-response elements (AREs) and a discriminating histone H3K4 methylation, likely contributes to this outcome between these two urological tumors. Further mechanistic studies indicated that AR-encoded circRNA-ARC1 might sponge/alter the availability of the miRNAs miR-125b-2-3p and/or miR-4736, to impact the metastasis-related PPARγ/MMP-9 signals to alter the PCa vs. BCa cell invasion. The preclinical study using the in vivo mouse model confirms in vitro cell lines data, showing that Enz treatment could increase PCa metastasis, which can be suppressed after suppressing circRNA-ARC1 with sh-circRNA-ARC1. Together, these in vitro/in vivo results demonstrate that antiandrogen therapy with Enz via targeting AR may lead to either increase PCa cell invasion or decrease BCa cell invasion. Targeting these newly identified AR/circRNA-ARC1/miR-125b-2-3p and/or miR-4736/PPARγ/MMP-9 signals may help in the development of new therapies to better suppress the Enz-altered PCa vs. BCa metastasis.

## Introduction

While the androgen receptor (AR) may play key roles to increase the tumor cell proliferation in several sex hormone-related tumors including prostate [1–6], bladder [7, 8], kidney [9, 10], breast [11], and liver [12–14], its impact on tumor cell invasion remains varied, as recent studies indicated that AR might function as a suppressor to decrease metastasis in prostate [1, 3, 4, 15–18] and liver [12–14] cancers, yet function as a stimulator to increase metastasis in bladder [7, 8, 19] and kidney [9, 10] cancers. The detailed mechanisms of these differential effects, however, remain unclear.

Circular RNAs (circRNAs), noncoding forms of RNA, are widely expressed in many tissues with distinct functions to influence the development of several diseases including tumor progression [20, 21]. Functions of circRNAs include gene regulation by competing with splicing of linear RNA [22], as templates of rolling circle amplification of RNA, and constraints on RNA folding, as well as acting as intermediates in RNA processing, or as sponges to influence microRNAs (miRNAs) availability in selective cells [23, 24].

The circRNAs are produced as a result of endogenous host gene transcription, and sequence analysis indicated that the AR locus can produce four circRNAs from a combination of exons. The linkage of these AR gene-coded circRNAs to the differential effects on the cell invasion of prostate cancer (PCa) vs. bladder cancer (BCa) in response to androgen-deprivation therapy (ADT) with the antiandrogen Enzalutamide (Enz) treatment (ADT-Enz), however, remains unclear.

Here we found that targeting androgens/AR with ADT-Enz or AR-shRNA led to increase AR-encoded circRNAs expression in PCa cells, yet suppress those circRNAs in BCa cells. The consequence of such differential alteration of the AR-encoded circRNAs may then increase PCa cell invasion, yet decrease BCa cell invasion. Further mechanistic analysis revealed that Enz/AR/AR-encoded circRNAs signaling might function via sponging/altering the expression of miR-125b-2-3p and/or miR-4736 to influence the metastasis-related PPARγ/MMP-9 signals to impact the PCa vs. BCa cell invasion.

## Results

### ADT with antiandrogen Enz or AR-shRNA differentially modulates the expression of the AR-encoded circRNAs

Early studies indicated that AR could enhance BCa metastasis [7, 8] yet suppress PCa metastasis [1, 3, 10, 15, 16]. The detailed mechanisms of why AR can play opposite roles to impact the metastasis of these two urological tumors, however, remain unclear.

**Fig. 1:**
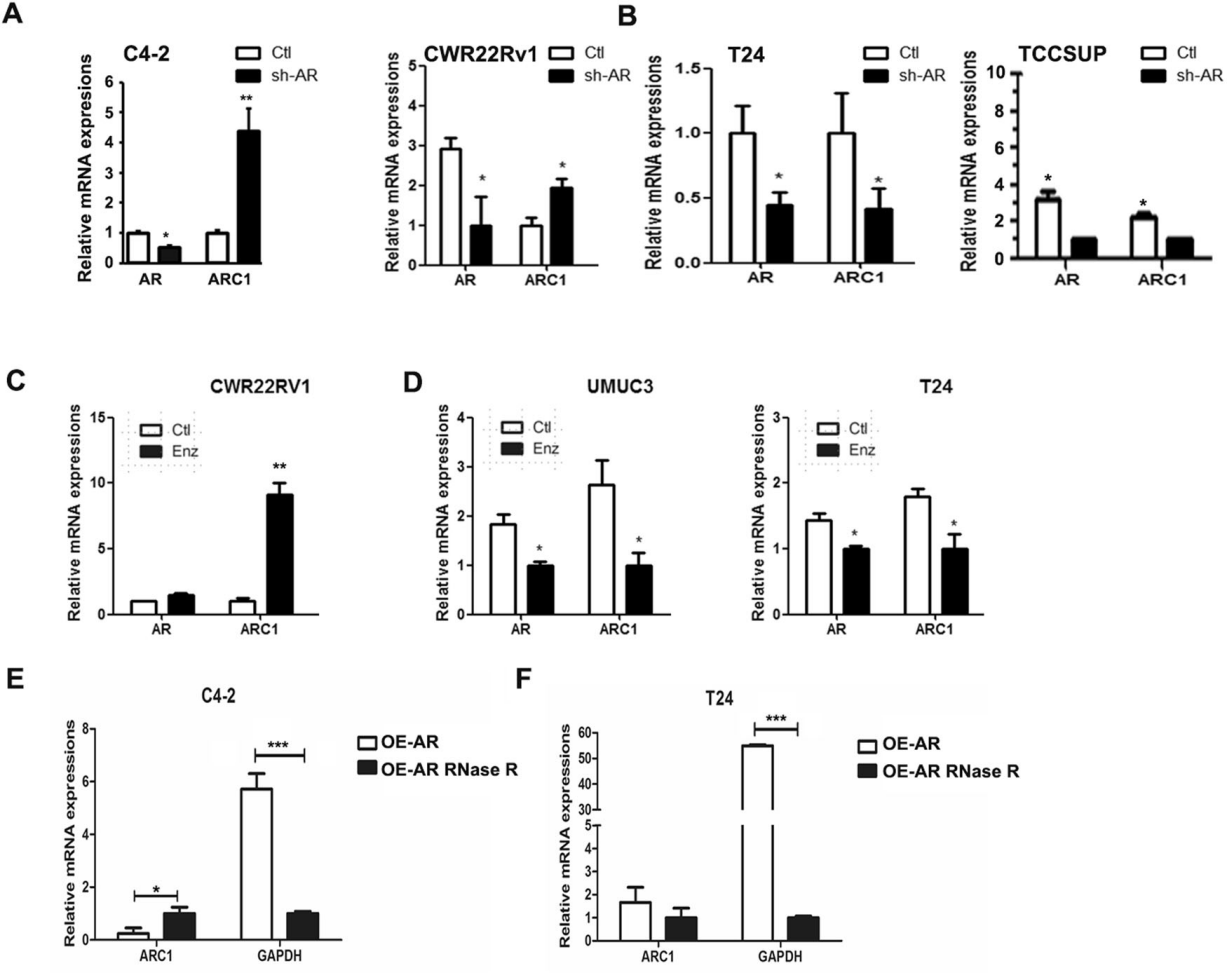
AR differentially regulates the expression of circRNAs in PCa vs. BCa cell lines. The qPCR analysis of circRNA-ARC1 (ARC1) in response to the knock down of AR in C4-2 and CWR22Rv1 cells (**A**), and in T24 and TCC-SUP cells (**B**). Effect of Enz treatment on expression of circRNA-ARC1 in PCa and BCa cell lines. The qPCR analysis of ARC1 expression after Enz treatment in CWR22Rv1 cell line (**C**) and in UMUC3 and T24 cells (**D**). RNase R-digestion assay to confirm circularity of ARC1 in C4-2 cells (**E**) and T24 cells (**F**). Data are presented as mean ± SEM. *P < 0.05, **P < 0.01, ***P < 0.001.

Here we first focused on the potential role(s) of AR-encoded circRNAs on the AR’s differential alteration of the PCa vs. BCa cell invasion, as these AR-encoded circRNAs are most closely related to the functional entity of AR. A database (Circbase) search indicated that the AR locus produces four circRNAs (Supplementary Fig. 1A, B). The circRNA-ARC1 was chosen for further study because it had smallest size and its circular property was confirmed by RNase R digestion. Interestingly, results revealed that targeting full-length AR (fAR) with AR-shRNA led to increase the expression of circRNA-ARC1 in castration-resistant PCa (CRPC) C4-2 and CWR22Rv1 cells (Fig. 1A). In contrast, targeting fAR with AR-shRNA led to an opposite effect of decreasing the expression of circRNA-ARC1 in BCa T24 and TCC-SUP cells (Fig. 1B). These contrasting results suggest that the differential AR function to increase vs. decrease cell invasion in these two urological tumors may involve differential expression of AR-encoded circRNA-ARC1.

To further substantiate the linkage between AR protein and AR-encoded circRNAs, we applied the 2nd approach using the recently developed antiandrogen Enz [25] to confirm the above results generated from AR-shRNA. As shown in Fig. 1C, adding Enz to suppress androgen/AR signaling led to increase the expression of circRNA-ARC1 in CRPC CWR22Rv1 cells, yet decrease the expression of circRNA-ARC1 in BCa T24 and UMUC3 cells (Fig. 1D).

Importantly, we applied the RNase R-digestion assay [26] to confirm the noncoding circRNA-ARC1 is indeed in the circular form as early studies demonstrated that RNase R, a highly processive 3′ to 5′ exoribonuclease, would not digest circRNAs due to a lack of a free 3′ end, yet it could digest linear RNAs with a 3′ single-stranded region of >7 nucleotides [27]. As shown in Fig. 1E, F, RNase R did not digest the circRNA-ARC1 while it can potently decrease the level of GAPDH mRNA in both C4-2 and T24 cells, suggesting that circRNA-ARC1 is indeed in the circular form.

Together, results from Fig. 1A–F suggest that targeting fAR with Enz or AR-shRNA can differentially alter the AR-encoded circRNA expression in PCa vs. BCa: increasing the circRNA-ARC1 expression in PCa cells, yet decreasing its expression in BCa cells.

### Mechanistic dissection of how targeting the AR can lead to increase circRNA-ARC1 expression in PCa cells, yet decrease its expression in BCa cells

**Fig. 2:**
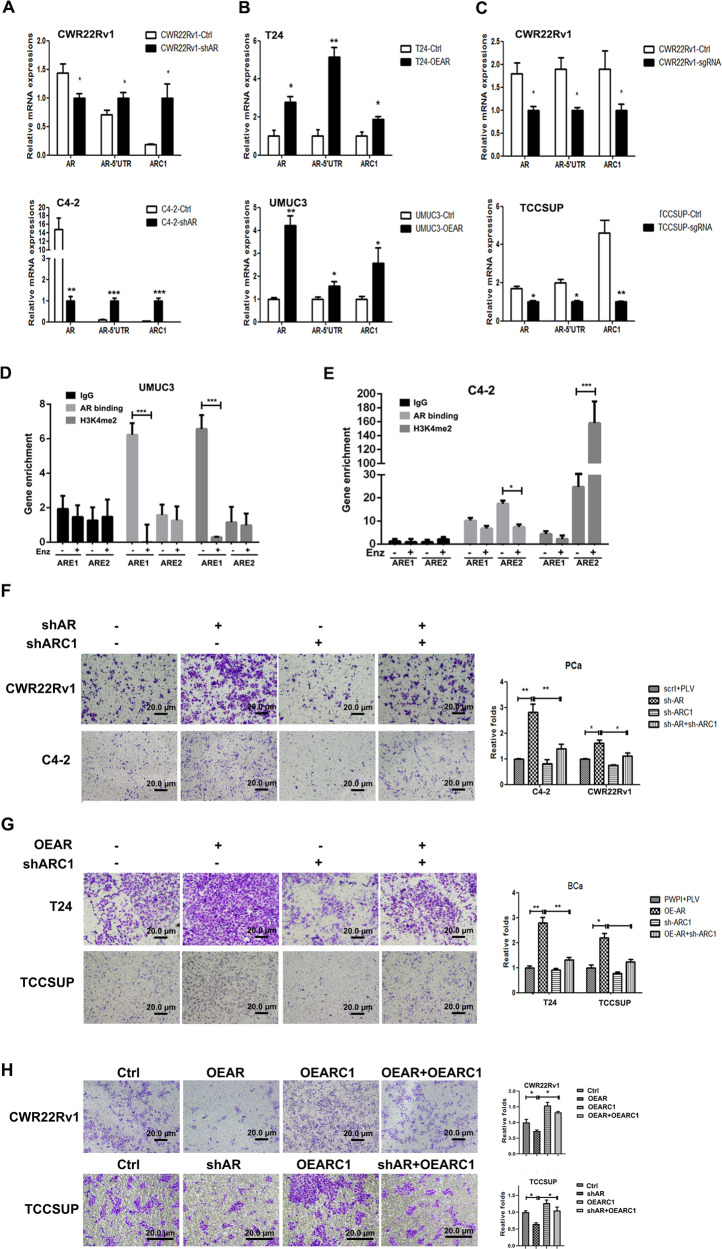
AR may influence PCa vs. BCa cell invasion via altering the circRNA-ARC1. **A** Endogenous transcripts of AR and circRNA-ARC1 (ARC1) as measured by primers for full-length AR and AR-5′UTR through semi-quantitative PCR assays in CWR22Rv1 and C4-2 cells in response to knocking down AR (shAR). **B** As in **A** qPCR assays in T24 and TCC-SUP cells with overexpressed AR (OEAR). **C** CRISPR-dCAS9-mediated transcriptional repression results in repression of ARC1 expression in PCa CWR22Rv1 and BCa TCC-SUP cells. (**D**–**E**) ChIP assay with AR antibody or H3K4me2 antibody of the two AREs in UMUC3 (**D**) and C4-2 (**E**) cells with and without Enz treatment. IgG served as negative control. **F** Knocking down ARC1 (shARC1)-suppressed cell invasion induced by knocking down AR (shAR) in CWR22RV1 and C4-2 cells. Invasion assay was performed in cells infected with the indicated lentiviral constructs. Representative microscopic photographs of invaded cells (original magnification, ×100) are shown in left panels. **G** Knocking down ARC1 (shARC1)-suppressed cell invasion induced by overexpression of AR (OEAR) in BCa T24 (upper panels) and TCC-SUP (lower panels) cells. Invasion assay was performed in cells that had been delivered with the indicated lentiviral constructs. Representative microscopic photographs of invaded cells (original magnification, ×100) were shown. **H** Overexpression of ARC1 can rescue cell invasion in PCa cells repressed by overexpression of AR in CWR22Rv1 (upper panels) cells and knocking down AR in BCa TCC-SUP (lower panels) cells. For **F**–**H**, the right panels indicate the quantitation of the cell invasion in the left panels. Scale bar: 20 µm. Data are presented as mean ± SEM. *P < 0.05, **P < 0.01, ***P < 0.001.

Since the AR-encoded circRNA-ARC1 is generated from transcription of the endogenous AR locus, we examined the relationship between the AR gene and circRNA-ARC1 expression, with *AR*-5′UTR as a gauge of endogenous AR transcription. As shown in Fig. 2A, decreasing AR via adding lentiviral AR-shRNA increased the *AR*-5′UTR in PCa CWR22Rv1 and C4-2 cells. In contrast, increasing AR via adding lentiviral AR-cDNA increased the *AR*-5′UTR in BCa T24 and UMUC3 cells (Fig. 2B), suggesting that AR-encoded circRNA-ARC1 expression is concordant with the endogenous AR transcription in response to manipulating the AR expression.

To further determine whether the differential expression of circRNA-ARC1 is linked with endogenous transcription of the *AR* locus, we applied the CRISPR-dCAS9-mediated transcriptional regulation [28] to examine the linkage of circRNA-ARC1 and its host gene-AR transcription. Results from transcriptional regulation from a combination of multiple sgRNAs targeting the promoter and enhancer of AR suggest that endogenous AR transcription is positively linked with circRNA-ARC1 production in PCa CWR22Rv1 and C4-2 as well as BCa TCC-SUP cells (Fig. 2C).

Together, results from Fig. 2A–C demonstrate that the differential alterations of the circRNA-ARC1 via targeting the AR are a result of differentially altering the endogenous AR transcription in PCa vs. BCa cells.

To further dissect the molecular mechanisms underlying the differential regulation of AR transcription in response to altering the AR, we focused on the AR binding to the two androgen-response elements (AREs), one (ARE1) located on the 1-kb upstream of AR gene and another (ARE2) located in the intron 3 [29]. We were interested to see if the autoregulation of AR may be different between PCa and BCa cells due to the differential binding of AR to the different AREs, so that circRNA-ARC1 was differentially expressed upon targeting the AR with Enz or shAR treatment. As shown in the Fig. 2D, a strong binding of AR to ARE1 was observed in the BCa UMUC3 cells, which could be released upon Enz treatment. However, instead of binding to ARE1, we found AR preferentially bound to ARE2 in the PCa C4-2 cells and this binding was diminished in the presence of Enz (Fig. 2E). Using histone H3 lysine4 methylation as an indicator of transcription activation to further dissect the mechanism, we found that H3K4me2 was highly enriched around ARE2, but not ARE1 in PCa C4-2 cells, and this enrichment was much more obvious upon Enz treatment, indicating a negative autoregulation of AR transcription in the PCa cells (Fig. 2E). In contrast, H3K4me2 enrichment was clearly observed around ARE1, but not ARE2, in the BCa UMUC cells, and this enrichment could be reduced in the presence of Enz, suggesting a positive autoregulation of AR transcription in BCa cells (Fig. 2D).

Together, results from Fig. 2A–E suggest that AR may play a positive autoregulation role in BCa via binding to ARE1 while a negative autoregulation role in PCa via binding to ARE2, and these opposing autoregulation roles may then result in increasing the circRNA-ARC1 expression in PCa cells, yet decreasing its expression in BCa cells.

### Differential expression of the circRNA-ARC1 led to AR’s differential impact on invasion in PCa vs. BCa cells

To study the consequences of differentially regulating the circRNA-ARC1 expression induced by targeting androgen/AR in the PCa vs. BCa, we applied the matrigel-coated transwell invasion assay, and results revealed that decreasing fAR (shAR) led to increase cell invasion in both PCa C4-2 and CWR22Rv1 cells (Fig. 2F), yet increasing fAR (OEAR) led to increase cell invasion in both BCa T24 and TCC-SUP cells (Fig. 2G).

Importantly, interruption approaches via adding shRNA to suppress the circRNA-ARC1 led to partially reverse the AR-shRNA-increased cell invasion in PCa C4-2 and CWR22Rv1 cells (Fig. 2F), while partially reversed the AR-increased cell invasion in BCa T24 and TCC-SUP cells (Fig. 2G). Furthermore, adding circRNA-ARC1 partially reversed the AR-decreased cell invasion in PCa CWR22Rv1 cells as well as reversed the AR-shRNA-decreased cell invasion in BCa TCC-SUP cells (Fig. 2H).

Together, results from Fig. 2F–H demonstrate that AR may decrease PCa cell invasion yet increase BCa cell invasion via altering the expression of AR-coded circRNA-ARC1.

### Mechanistic dissection of how circRNA-ARC1 can influence the PCa vs. BCa cell invasion: via sponging/altering the miR-125b and/or miR-4736

Next, to dissect the mechanism of how AR-modulated circRNA-ARC1 can influence the PCa vs. BCa cell invasion, we focused on miRNAs, since early studies indicated that circRNAs might function as a miRNAs sponge or miRNAs reservoir to alter the breast or colorectal tumor progression [30].

Through bioinformatics analysis of existing databases (DIANA miRGen, MicroCosm Targets, RNA22, and RegRNA2.0) and published literatures, we found that circRNA-ARC1 might be able to interact with several miRNAs (see Supplementary Fig. 2A, B). Among these potential candidate miRNAs, we decided to focus on miR-125b-2-3p and miR-4736 since recent studies indicated that these two miRNAs might play key roles to alter the progression of BCa [31], PCa [32, 33], and spinal chordoma [34].

**Fig. 3:**
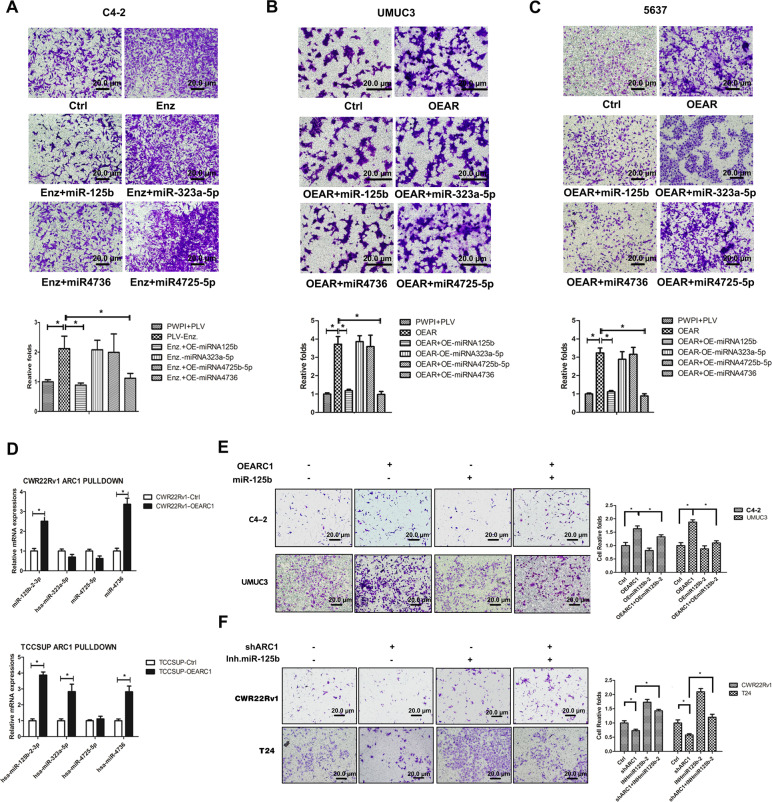
AR-circRNA-ARC1 regulates PCa vs. BCa cell invasion through miR-125b-2-3p and/or miR-4736. **A** The miRNAs can repress cell invasion induced by Enz in PCa C4-2 cells. Invasion assay was performed in C4-2 cells transduced with the indicated lentiviral constructs with Enz treatment (10 μM, 48 h). The miRNAs can repress cell invasion induced by overexpression of AR in BCa UMUC3 (**B**) and TCC-5637 (**C**) cells. Invasion assays were performed in BCa cells transduced with the indicated lentiviral constructs. For **A–C** top panels, representative microscopic photographs of invaded cells (original magnification, ×100) were shown, with lower panels quantitation of images (cell numbers were counted in six randomly chosen microscopic fields per membrane). Scale bar: 20 µm. **D** The circRNA-ARC1 (ARC1) can interact with miRNA-125b-2-3p and miR-4736 in PCa and BCa cells. ARC1 was pulled-down on the streptavidin beads by biotinylated oligonucleotide complimentary to the fusion junction of the circRNA-ARC1. The miRNAs associated with the ARC1 were determined through quantitative PCR of the respective miRNAs in PCa CWR22Rv1 and BCa TCC-SUP cells with or without overexpression of ARC1. **E** miR-125b-2-3p can suppress ARC1-induced cell invasion in PCa C4-2 (upper panels) and BCa UMUC3 (lower panels) cells. **F** miRNA-125b-2-3p inhibitor can rescue cell invasion repressed by ARC1-knocked down in PCa CWR22Rv1 (upper panels) and BCa T24 (lower panels) cells. For **E**, **F** left panels are representative microscopic photographs of invaded cells and right panels the quantitation of cell invasion. Scale bar: 20 µm. Data are presented as mean ± SEM. *P < 0.05.

Indeed, we found that Enz treatment increased PCa cell invasion was reversed/blocked after adding miR-125b-2-3p or miR-4736 in PCa C4-2 cells (Fig. 3A). In contrast, we found that adding miR-125b-2-3p or miR-4736 to BCa UMUC3 and TCC-5637 cells reversed/blocked the AR-increased BCa cell invasion (Fig. 3B, C). The choice of these two miRNAs was also supported by the failure of miRNA-323a-5p and -4725-5p to block Enz’s effect.

Together, results from Fig. 3A–C suggest that miR-125b-2-3p and miR-4736 may play key roles to mediate the circRNA-ARC1-modulated PCa vs. BCa cell invasion.

To examine the molecular basis of the functional interaction between miRNAs and circRNA-ARC1, we applied the pull-down assay to isolate the circRNA-ARC1 via the biotin-labeled oligonucleotide that is complimentary to the fusion junction of the circRNA-ARC1 and measured its associated miRNAs. The results revealed that circRNA-ARC1 could function as a sponge to absorb/interact with miR-125b-2-3p and miR-4736 in both PCa CWR22Rv1 cells and BCa TCC-SUP cells (Fig. 3D).

Importantly, results from the rescue assay revealed that adding miR-125b-2-3p led to partially reverse/block the circRNA-ARC1-increased cell invasion in PCa C4-2 and BCa UMUC3 cells (Fig. 3E), yet adding miR-125b-2-3p inhibitor partially reversed/blocked the circRNA-ARC1-shRNA-suppressed cell invasion in PCa CWR22Rv1 and BCa T24 cells (Fig. 3F).

Together, results from Fig. 3A–F suggest that AR-modulated circRNA-ARC1 may alter the PCa vs. BCa cell invasion via sponging/interacting with/altering the miR-125b-2-3p and miR-4736 to further influence the target genes related to tumor cell invasion.

### Mechanistic dissection of how circRNA-ARC1/miR-125b-2-3p/miR-4736 signaling can alter the PCa vs. BCa cell invasion: via modulating the PPARγ/MMP-9 signals

To further dissect the molecular mechanisms of how AR-modulated circRNA-ARC1/miR-125b-2-3p/miR-4736 signaling can alter the PCa vs. BCa cell invasion, we surveyed those genes related to PCa vs. BCa metastasis [35, 36] and their potential linkages to these miRNAs. Such analyses led us to focus on the PPARγ/MMP-9 signals, as their downstream targets (PPARGC1B and PDK1) are linked to our identified miRNAs [37–40].

**Fig. 4:**
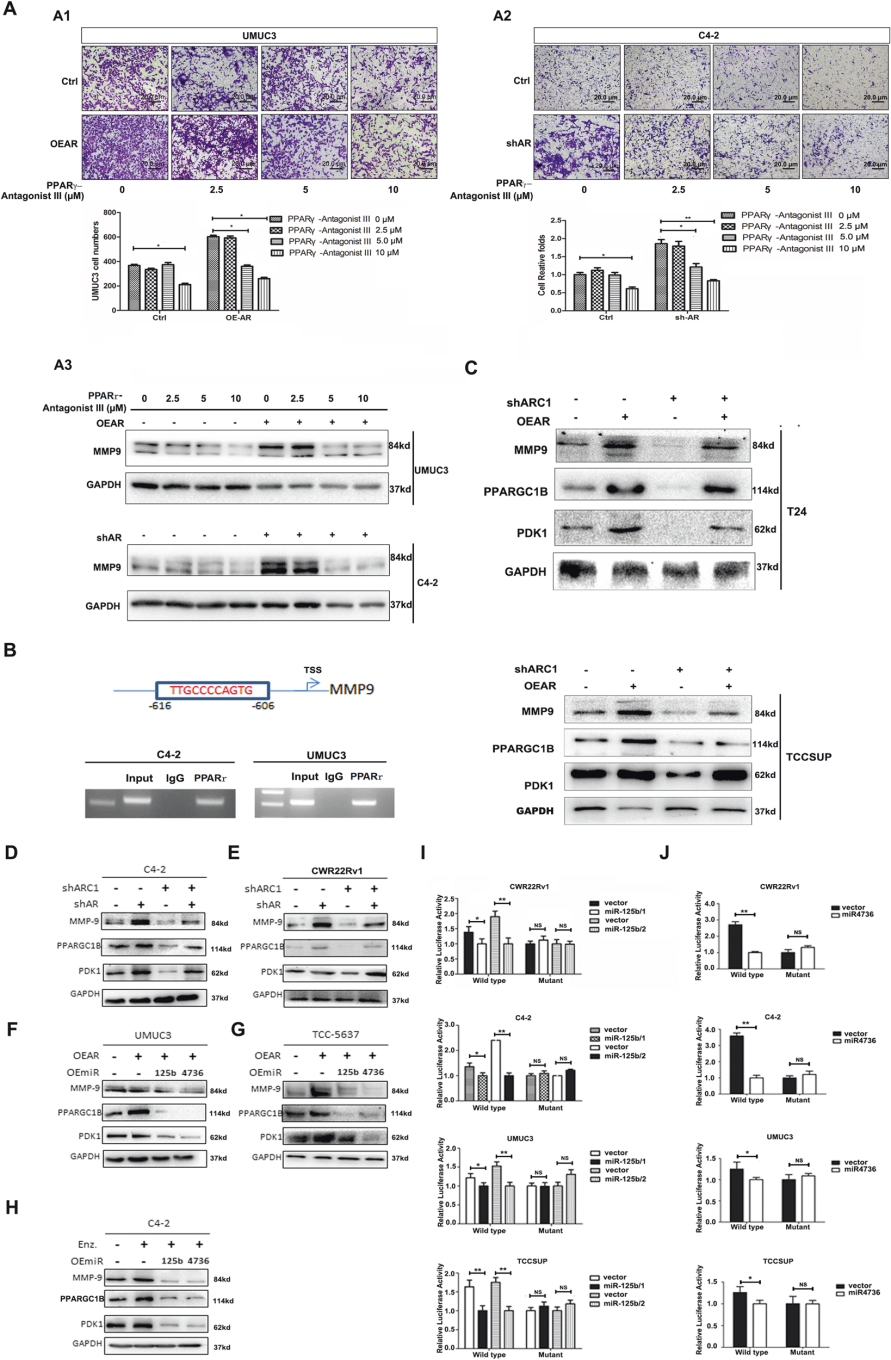
AR-modulated circRNA-ARC1/miR-125b-2-3p/miR-4736 signals regulated PCa vs. BCa cell invasion via altering the PPARGC1B/PDK1/MMP-9 signals. Treating with PPARγ antagonist III can suppress cell invasion induced by overexpression of AR in BCa UMUC3 cells (**A1**) and knocking down AR in PCa C4-2 cells (**A2**). Invasion assays were performed in cells that had been delivered with the indicated lentiviral constructs and treated with different concentrations of the antagonist. Top panels, representative microscopic photographs of invaded cells (original magnification, ×100) were shown. Bottom panels, the quantitation of cell invasion in **A1, A2**. Scale bar: 20 µm. **A3** MMP-9 expression was regulated by AR expression and PPARγ antagonist in BCa UMUC3 (upper panels) and PCa C4-2 (lower panels) cells. **B** MMP-9 promoter analysis using online software ALGGEN PROMO. The top panel indicates that there is a PPARγ binding element (PPARγRE) upstream of MMP-9 transcription start site (TSS). The lower panel indicates the results of chromatin immunoprecipitation (ChIP) results in PCa C4-2 (left) and BCa UMUC3 (right) cells. **C** Knocking down circRNA-ARC1 (ARC1) can suppress expression of MMP-9, PPARGC1B, and PDK1 induced by overexpression of AR in BCa T24 (upper panels) and TCC-SUP (lower panels) cells. Knocking down ARC1 can suppress expression of MMP-9, PPARGC1B, and PDK1 induced by knocking down AR in PCa C4-2 (**D**) and CWR22Rv1 (**E**) cells. The miR-125b-2-3p and miR-4736 can suppress expression of MMP-9, PPARGC1B, and PDK1 induced by overexpression of AR in BCa UMUC3 (**F**) and TCC-5637 (**G**) cells. **H** The miR-125b-2-3p and miR-4736 can suppress expression of MMP-9, PPARGC1B, and PDK1 induced by Enz in PCa C4-2 cells. **I**, **J** The miRNA-125b-2-3p modulates reporter activity of 3′UTR of PPARGC1B/PDK1. Normalized luciferase activity was measured after transfection of wild-type or mutant PPARGC1B/PDK1 3′UTR reporter construct in cells transduced with the indicated lentiviral constructs for miR-125b-2-3p or miR-4736 in PCa (**I**) and BCa (**J**) cells. Data are presented as mean ± SEM. *P < 0.05, **P < 0.01, and NS = no significant difference.

To test the role of PPARγ/MMP-9 signals in this process, we first treated cells with the PPARγ antagonist for the transwell invasion assay, and results revealed that AR-altered cell invasion was particularly sensitive to the PPARγ-antagonist III in BCa UMUC3 cells and PCa C4-2 cells (Fig. 4A1, A2). Consistent with these results, adding PPARγ-antagonist III also partially reversed AR-increased MMP-9 expression in BCa UMUC3 and PCa C4-2 cells (Fig. 4A3).

Together, results from Fig. 4A1–A3 suggest that AR may function via altering the PPARγ/MMP-9 signals to modulate the PCa vs. BCa cell invasion.

To further dissect the mechanism of how PPARγ can modulate the MMP-9 at the molecular level, we identified one potential PPARγ-response element (PPARγRE) via PROMO website in the 1-kb 5′ promoter region of the MMP-9 gene (Fig. 4B). We then used the chromatin immunoprecipitation (ChIP) in vivo binding assay to detect PPARγ binding to this potential PPARγRE, and revealed that PPARγ could bind to the PPARγRE located in the 5′ promoter region of MMP-9 (Fig. 4B), suggesting that PPARγ may directly regulate MMP-9 expression via regulating PPARγ binding to the PPARγRE.

Consistent with the direct regulation of the MMP-9 by PPARγ, we also confirmed that increasing AR via adding AR-cDNA in both BCa T24 and TCC-SUP cells significantly increased the expression of MMP-9 (Fig. 4C). In contrast, decreasing AR via adding AR-shRNA increased the expression of MMP-9 in PCa C4-2 and CWR22Rv1 cells (Fig. 4D, E). Importantly, AR-induced MMP-9 in BCa cells or shAR-increased MMP-9 in PCa cells could be partially blocked by the altering circRNA-ARC1 expression (Fig. 4C–E).

Together, results from Fig. 4B–E suggest that PPARγ may transcriptionally regulate the MMP-9 expression via direct binding to the PPARγRE on its 5′ promoter region.

To further demonstrate that AR-modulated circRNA-ARC1 may function via sponging/altering the miR-125b-2-3p/miR-4736 to regulate PPARγ/MMP-9 signals, we applied the interruption approach, and results revealed adding miR-125b-2-3p or miR-4736 led to reverse the AR-enhanced expression of PPARGC1B and PDK1 as well as MMP-9 in BCa cells (Fig. 4F, G). Similar results were also obtained showing that exogenous miR-125b-2-3p/miR-4736 in PCa C4-2 cells led to reverse the Enz-increased PPARGC1B/PDK1-MMP-9 expression (Fig. 4H).

It is worth noting that adding either miR-125b-2-3p or miR-4736 can result in repression of both PDK1 and PPARGC1B expression to a different degree in both PCa and BCa cells. Indeed, a survey of potential miRNA target genes with a relaxed criterion in target selection in the database of Targetscan indicated that both miRNAs can target both genes albeit with different predictive stringencies.

Interestingly, human clinical sample surveys using TCGA datasets revealed that AR has a positive correlation with PPARγ, PDK1, and PPARGC1B (Supplementary Fig. 3A–C).

Together, results from Fig. 4A–H suggest that Enz/AR may differentially impact the PCa vs. BCa cell invasion via altering the AR-modulated circRNA-ARC1/miR-125b-2-3p/miR-4736/PPARγ/MMP-9 signals.

### Mechanistic dissection of how AR/circRNA-ARC1/miR-125b-2-3p/miR-4736 axis can alter the PPARGC1B/PDK1 expression: via direct binding to its 3′UTR mRNA

To dissect the molecular mechanism of how miR-125b-2-3p can modulate the PPARGC1B expression, we first identified two potential miRNA target sequence in the 3′UTR mRNA of PPARGC1B. We then inserted 915 and 1196-bp fragments from the PPARGC1B 3′UTR mRNA with the predicted miR-125b-2-3p target site into a dual-luciferase reporter psiCHECK™-2 downstream of the Renilla luciferase ORF, as well as prepared a mutated version at the predicted target site.

As expected, the Luciferase Assay results revealed that expression of miR-125-2-3p markedly decreased luciferase activity in PCa CWR22Rv1 or C4-2 cells as well as BCa UMUC3 or TCC-SUP cells transfected with the wild-type PPARGC1B 3′UTR, but not the mutant PPARGC1B 3′UTR (Fig. 4I).

Similar results were also obtained when we replaced miR-125-2-3p with miR-4736 binding to the wild-type PDK1 3′UTR via insertion of the 972-bp fragment from the PDK1 3′UTR with the predicted miR-4736 target site into the psiCHECK™-2 vector as well as a mutated version at the predicted target site in the same PCa and BCa cells (Fig. 4J).

Together, results from Fig. 4I, J suggest that miR-125b-2-3p/miR-4736 can directly regulate PPARGC1B/PDK1 expression via direct binding to their 3′UTRs mRNA.

### Preclinical study using the in vivo mouse model to prove circRNA-ARC1 roles in the Enz-induced PCa metastasis

To validate the above results from in vitro studies of PCa and BCa cell lines in vivo, we applied an orthotopic PCa xenograft mouse model. We generated CWR22Rv1 cells with luciferase expression with added sh-circRNA-ARC1 (Groups 3 and 4) to suppress circRNA-ARC1 expression or with vector control (Groups 1 and 2) and implanted these cells into the anterior prostate lobes of nude mice (*n* = 32 for each group), with (Groups 2 and 4) or without (Groups 1 and 3) intra-peritoneal (i.p) injection of Enz (30 mg/kg). Tumor growth and metastases were evaluated weekly with the In Vivo Imaging Systems (IVIS) analysis in a noninvasive manner.

**Fig. 5:**
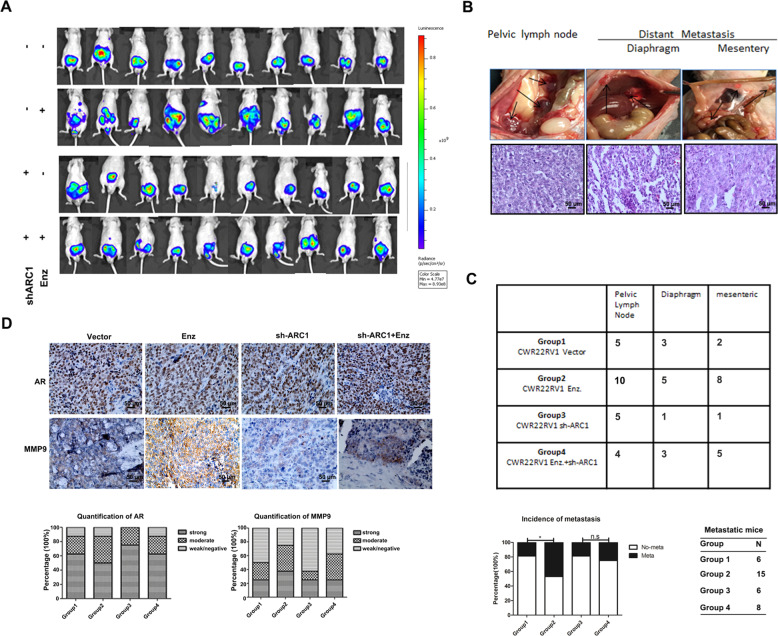
In vivo mice studies confirmed the role of Enz and ARC1 in PCa metastasis. **A** IVIS imaging was used to determine the metastasis in each group of mice as indicated. The images were obtained at different times with the same exposure and digitally arranged. **B** Representative images of pelvic lymph node, diaphragm, mesentery metastasis (upper panesl) with IHC staining in lower panels. **C** Tabulation of mice with metastasis. Metastatic site tabulations were listed. Chi-square test was used to analyze the results (upper panel), Enz significantly promoted CWR22Rv1 PCa metastasis (15 mice in Enz treated group (*n* = 32) vs. 6 mice in vehicle-treated group (*n* = 32), *P* = 0.015) and Enz failed to promote tumor metastasis when circRNA-ARC1 was knocked down (lower panel). The Pearson’s chi-square test shows significant differences among all four groups (*P* value = 0.0349). **D** H&E staining confirmed the tumor tissue and representative images of IHC staining for AR and MMP-9 with quantitations (lower panels) on the right as mean ± SEM. *P < 0.05 and ns = no significant difference.

After 6 weeks, IVIS imaging showed that treating with Enz led to increase the PCa metastasis (Fig. 5A, Group 2 vs. Group 1). Importantly, Enz failed to promote PCa metastasis when circRNA-ARC1 expression was suppressed with sh-circARC1 (Fig. 5A, Group 3 vs. Group 4). Similar results were also obtained showing that suppressing circARC1 expression led to decrease the metastatic luciferase signals upon Enz treatment (Fig. 5A, Group 4 vs. Group 2). We also confirmed the IVIS assays with direct measurement of the metastatic tumor growth with the H&E staining in pelvic lymph nodes and the diaphragm (Fig. 5B, C).

Importantly, our IHC staining from these PCa xenografts demonstrated that suppressing circARC1 led to decrease the MMP-9 expression compared to that from the control group, consistent with our in vitro findings. We also found that there was no significant difference of AR expression among different groups (Fig. 5D).

**Fig. 6:**
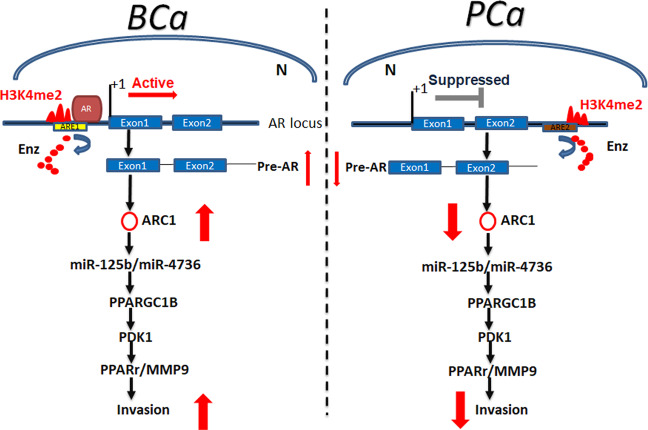
Schematic depiction that AR and Enz could differentially regulate cell invasion of BCa and PCa via differential modulation of the circRNA-ARC1/miRNAs/PPARGC1B/PDK1/MMP-9 signals. In BCa, AR binds ARE1 (located in the 5′ upstream of AR locus) and promotes the generation of pre-AR and ARC1 by activating AR transcription. In PCa, AR prefers to bind ARE2 (located in the intron 2 of AR locus) and decreases the production of pre-AR and ARC1 by suppressing AR transcription. Enzalutamide (Enz) prevents AR from binding ARE1 in BCa and ARE2 in PCa. ARC1 increases the expression level of PPARGC1/PDK1/MMP-9 signaling by sponging miR-125b and miR-4736, leading to enhanced cell invasion. High level of H3K4me2 serves as one biomarker of gene transcription activation. N nucleus.

Together, results from in vivo mouse model studies in Fig. 5A–D are in agreement with the in vitro cell lines studies and demonstrated that targeting the circRNA-ARC1 could suppress the Enz-increased PCa metastasis via altering the circRNA-ARC1/miR-125b-2-3p/miR-4736/PPARγ/MMP-9 signals (see outline in Fig. 6).

## Discussion

PCa is a leading cause of cancer death among males in western countries, and increasingly so in the developing countries like China [41, 42]. BCa ranks the highest among urinary tumors in the world with a clear male dominance in incidence [43]. Recent studies also indicated that AR might play positive roles to promote tumor cell proliferation in both PCa and BCa cells [44]. However, as shown in this study, AR may also play opposite roles to increase BCa cell invasion, yet suppress PCa cell invasion.

The circRNAs are the non-encoding RNAs that are mainly located on the exons, but can also occur in any genomic region, with 85% of circRNAs aligned in sense orientation to known protein-coding genes that can span 1–5 exons [24, 45]. The circRNAs are likely more stable than linear RNAs as a result of inherent higher resistance to nuclease activity. In addition, circRNAs can also function as a natural sponge of miRNAs [46–48] to link their importance in the disease regulation [24, 29, 30], including anti-cancer effects in malignant melanoma tumors [49].

Our findings here that ADT-Enz (or AR-shRNAs) may function via differentially altering the AR-encoded circRNA-ARC1 to increase PCa metastasis, yet decrease BCa metastasis, not only may help treatment to improve the ADT-Enz therapy efficacy via reducing those adverse effects, it may also represent the first evidence to link AR-encoded circRNAs to the differential anti-AR therapies. Interestingly, a recent study also found that AR might suppress its transcription in a LSD1-dependent manner [50], and ADT-Enz therapy may de-repress the AR transcription, which can then result in increasing the AR transcription to produce more AR protein to function in the castration environment with lower androgen levels [51]. Here we found that, in addition to AR protein encoded by the *AR* locus, the *AR* transcription could also result in generating additional molecules, like circRNAs, to play roles via sponging the miRNAs to regulate cell invasion and likely tumor metastasis. It will be interesting in the future studies to see if ADT-Enz may also function via differentially regulating AR-encoded circRNAs to further impact those ADT-Enz-induced adverse effects that involve altering either GR signals [52], AR-coactivator NCOA2 signals [3], or TGFß1 signals [53].

The precise mechanisms at the molecular level of how differential complexes may guide AR to bind to the distinct AREs located on different regions of the AR gene remain unclear. Our data showed that AR preferred to bind to the ARE1 in BCa cells, yet preferred to bind to the ARE2 in PCa cells. These differential bindings of AR to the different AR loci resulted in a positive autoregulation in BCa cells vs. a negative autoregulation in PCa cells, which was monitored by H3K4me2 levels. It is likely the tumor origin of tissues as well as cellular signaling networks in the PCa vs. BCa are different so that AR may associate with different co-factors/co-repressors to form distinct complexes that result in binding to different AREs in the AR locus, and consequences of such differential binding may then result in a positive autoregulation in BCa cells vs. a negative autoregulation in PCa cells. Indeed the expression of AR protein in PCa vs. BCa cells is also different with much higher AR protein detected in the PCa cells.

The human clinical data indicated that ADT is an effective therapy in suppressing the recurrence of AR-positive BCa cells [54], with its therapeutic durability in BCa up to 2–3 years. In PCa, results from a recent NGS effort in a large cohort of PCa samples suggested that constitutively active AR splice variants may be linked to the cell invasion in 40–50% of CRPC patients [55]. It will be interesting in the future to see if targeting this newly identified AR/circRNA-ARC1/miR-125b-2-3p/miR-4736/PPARγ/MMP-9 signaling with small molecules (for example, shRNA of circRNA-ARC1 as shown in our in vivo mice data) may help in the development of novel therapeutic approaches to increase the Enz efficacy with less adverse effects to better suppress metastases in patients with PCa and BCa.

## Materials and methods

### Cell culture and transfection

The human PCa cell lines, C4-2 and CWR22Rv1, the BCa cell lines, T24, TCC-SUP, UMUC3, TCC-5637, and 293T, were originally purchased form American Type Culture Collection (ATCC, Manassas, VA). All PCa cells were cultured in RPMI 1640. All BCa cells were cultured in DMEM, all the media contained 10% FBS, penicillin (25 units/ml), and streptomycin (25 µg/ml) while cells were cultured in the humidified 5% CO_2_ environment at 37 °C.

To generate AR overexpressing or AR knocked-down stable cell populations, C4-2, CWR22Rv1, T24, TCC-SUP, UMUC3, or TCC-5637 cells were infected with lentiviral vectors, pWPI-AR/pWPI-Vec or pLKO1-sh-AR/pLKO1-scr. The lentiviruses were produced in 293T cells with the psAX2 packaging plasmid and pMD2G envelope plasmid together with the transfer plasmid. After 48h transfection, virus supernatants were collected, concentrated, and frozen at −80 °C for later use. Enz and PPARγ-Antagonist III treatments were performed by culturing cells with 10 µM of each drug for 48 h.

The transient transfection was performed using the Lipofectamine 3000 (Invitrogen) reverse transfection protocol according to the manufacturer’s instructions.

### Reagents and materials

GAPDH (6c5), AR (N-20) antibodies, and PPARγ-Antagonist III were purchased from Santa Cruz Biotechnology. PPARγ, PDK1, PGC-1β, and MMP-9 antibody were purchased from One World Lab. Anti-mouse/rabbit secondary antibody for Western Blot was from Invitrogen. Normal rabbit IgG was also from Santa Cruz Biotechnology. The PPARγ-Antagonist III and Enz stock concentrations were 10 mM.

### Test of RNase R resistance

Total RNA was isolated by TRIZOL lysis followed by PureLink purification of the aqueous phase (Life Technologies). Total RNA at 2 μg was treated in a 10-μl reaction with 0 units (mock treatment) or 20 units of RNase R (Epicentre) in 1×RNase R buffer, 1-unit/μl murine Ribonuclease Inhibitor (New England Biolabs), and incubated at 37 °C for 1 h. Then 1 μl 1-mM EDTA, 1 μl 10-mM each dNTP, and 1 μl 100-µM random hexamer were added and the RNA denatured at 65 °C for 5 min and placed on ice. Then 4-μl 10X buffer (250 mM Tris-HCl/pH 8, 125 mM KCl, and 15 mM MgCl_2_), 1-μl murine Ribonuclease Inhibitor (40 units/μl), and 1-μl Superscript III (Life Technologies) were added and this cDNA reaction was incubated at 25 °C for 10 min, 50 °C for 50 min, 55 °C for 10 min, and 85 °C for 5 min. Then 0.5-μl cDNA reaction was used as the template for qPCR and fraction resistance was computed as 2^∧^(RNase R Ct - Mock Ct).

### CircRNA-ARC1 overexpression plasmid construction

The pWPI-circARC1 was constructed by PCR amplifying the circRNA locus, including 1-kb upstream and 200-bp downstream to the nonlinear splice sites. The PCR fragment was amplified and ligated with Pme I + Pac I digested pWPI (Addgene) using the Gibson ligation system. PLKO-shRNAs were constructed following the standard protocol. Briefly, oligos were annealed and ligated with EcoR I + Age I digested PLKO vector. The junction of circARC1 was used as a template to design shRNAs.

### Lentivirus packaging

The pLVTHM-miR-125b-2-3p, pLVTHM-miR-4736, pLVTHM-miR-4725, pWPI-OE-ARC1, pLVTHM-shARC1, or pLKO.1-shAR, the psAX2 packaging plasmid, and pMD2G envelope plasmid were transfected into 293T cells using the standard calcium-chloride transfection method for 48 h to get the lentivirus supernatants. The lentivirus supernatants were collected and concentrated by density gradient centrifugation, then frozen in −80 °C for later use.

### RNA extraction, miRNA extraction, and reverse transcription and quantitative real-time PCR (qRT-PCR) analysis

For RNA extraction, total RNAs were isolated using Trizol reagent (Invitrogen) and 1 µg of total RNA was subjected to reverse transcription using Superscript III transcriptase (Invitrogen). The qRT-PCR was conducted using a Bio-Rad CFX96 system with SYBR green to determine the mRNA expression level of a gene of interest. Expression levels were normalized to the expression of TATA box binding protein TBP RNA. Primers used for genes of interest were listed in Supplementary Table S1. The miRNAs were also reversed transcribed from total RNA. Briefly, 1 µg of total RNA was processed for poly A addition by adding 2 units of polymerase with 1-mM ATP in 1x RT buffer at 37 °C for 20 min in 10-μl volume, then adding 50-pmol anchor primer to 11 μl, incubating at 65 °C for 5 min, then 4 °C for 2 min. For the last step of cDNA synthesis, we added 2-µl 5x RT buffer, 2 µl 10-mM dNTP, 1-µl reverse transcriptase to total 20 µl, and incubated at 42 °C for 1 h. qRT-PCR was conducted using a Bio-Rad CFX96 system with Tagman probe to determine the miRNA expressions. Expression levels were normalized to the expression of 5s RNA and/or U6.

#### Western blot analysis

Cells were lysed in RIPA buffer, and proteins (30 µg) were separated on 8–10% SDS/PAGE gel and then transferred onto PVDF membranes (Millipore, Billerica, MA). After blocking membranes, they were incubated with appropriate dilutions of specific primary antibodies, and then incubated with horseradish peroxidase (HRP)-conjugated secondary antibodies and visualized using the ECL system (Thermo Fisher Scientific, Waltham, MA). The antibodies used were listed in Supplementary Table S2.

#### Cell invasion assay

The invasion capability of PCa and BCa cells was determined using the Transwell assay. Briefly, for invasion assay, before seeding the cells, 100 µl of Matrigel (BD, Inc., Franklin Lakes, NJ.) was dissolved in 1.5-mL serum-free DMEM and applied to upper chambers of 8-µm-pore-size polycarbonate membrane filters (Corning, Inc., Corning, NY), and the transwells were incubated at 37 °C for 5 h. Harvested cells were then seeded with serum-free media into the upper chambers at 1 × 10^5^ cells/well, and the bottom chambers contained mixed conditioned media (CM) with 10% FBS, and then incubated for 24 h at 37 °C. Following incubation, the invaded cells attached to the lower surface of the membrane were fixed by 4% paraformaldehyde and stained with 1% crystal violet. Cell numbers were counted in five randomly chosen microscopic fields (100×) per membrane.

#### Luciferase Assay

The human PPARGC1B/PDK1 3′UTR containing wild-type or mutant miRNA-responsive elements were cloned into the psiCHECK™-2 vector construct (Promega, Madison, WI) downstream of the Renilla luciferase ORF. Luciferase activity was measured by the Dual-Luciferase Assay (Promega) according to the manufacturer’s manual.

#### Chromatin Immunoprecipitation Assay (ChIP)

Cells were cross-linked with 4% formaldehyde for 10 min followed by cell collection and sonication with a predetermined power to shear chromatin to 200–500-bp DNA fragments. Then, cell lysates were pre-cleared sequentially with normal rabbit IgG and protein A-agarose. Anti-PPARγ antibody was used for immunoprecipitation. For the negative control, IgG was used in the reaction. Specific primer sets were designed to amplify a target sequence within the human MMP-9 promoter, and PCR products were identified by agarose gel electrophoresis.

#### Pull-down Assay

The culture cells were lysed in RIPA lysis buffer (20-nM Tris-HCl/pH 7.5, 150-mM NaCl, 1-mM Na_2_ EDTA, 1-mM EGTA, 1% NP-40, 1% sodium deoxycholate, 2.5-mM sodium pyrophosphate, 1-mM beta-glycerophosphate, 1-mM Na_3_VO_4_, and 1-µg/ml leupeptin) supplemented with RNase inhibitor (1.5 µl). The supernatant was incubated with 250 nM of anti-sense oligos overnight at 4 °C. Streptavidin Agarose beads were incubated with the supernatant for 2 h. The complex was centrifuged at 3000 rpm, and the beads were washed five times with RIPA lysis buffer. The RNA was extracted using Trizol (Invitrogen) according to the manufacturer’s protocol and subjected to RT-PCR analysis.

#### CRISPR-dCAS9-mediated transcriptional regulation

The cultured cells were first transfected with shAR and PWPI plasmid, then CRISPR-dCas9-CRAB plasmids and sgRNA targeting the host gene-AR expression vector were simultaneously infected into cells. The cells were harvested at 72 h after transfection. The RNA was extracted as above using Trizol (Invitrogen) according to the manufacturer’s protocol and subjected to RT-PCR analysis.

#### In vivo studies

Six-to-eight weeks old nude mice were purchased from NCI and divided into four treatment groups (*n* = 32 for each group): (1) Vector (DMSO); (2) Enz; (3) pWPI+shARC1; and (4) Enz+shARC1. The transduced CWR22Rv1 cells suspended in media were mixed with Matrigel (1:1, v/v) and injected into the anterior prostates (1 × 10^6^ cells/AP) of these nude mice. Tumor development and metastasis were monitored by Luminescence Imager (IVIS Spectrum, Caliper Life Sciences, Hopkinton, MA) once a week. When the tumors become palpable 2 weeks after implantation, the shARC1 and vector mice were randomly assigned into two groups, respectively (32 mice per group), receiving various therapies by i.p. injection 3 times/wk/4 wks (Enz at 30 mg/kg or DMSO every other day). The mouse body weights were monitored weekly as well as tumors by the IVIS system for another 2–3 weeks. At the end of the treatment, mice were sacrificed and tumor growth and metastases at lymph nodes as well as to distant organs were analyzed. The animal experiments were approved and supervised by the University Committee on Animal Resource of University of Rochester Medical Center.

#### H&E and immunohistochemical (IHC) staining

Tissues were fixed in 10% (v/v) formaldehyde in PBS, embedded in paraffin, and cut into 4-μm sections and used for H&E staining and IHC staining with specific primary antibodies against AR/MMP-9. To enhance antigen exposure, the slides were treated with 1 × EDTA at 98 °C for 10 min for antigen retrieval. The slides were incubated with endogenous peroxidase blocking solution, and then were incubated with the primary antibody at 4 °C overnight. After rinsing with Tris-buffered saline, the slides were incubated for 45 min with biotin-conjugated secondary antibody, washed, and then incubated with enzyme conjugate HRP-streptavidin. Freshly prepared DAB (Zymed, South San Francisco, CA) was used as substrate to detect HRP. Finally, slides were counter-stained with hematoxylin and mounted with aqueous mounting media. Positive cells were calculated as the number of immunopositive cells × 100% divided by total number of cells/field in ten random fields at ×400 magnification.

#### Statistical analysis

Data were expressed as mean ± SEM from at least three independent experiments with each data point in triplicate. Statistical analyses involved the Tukey’s test with SPSS 17.0 (SPSS Inc., Chicago, IL). Chi-square test was used to analyze the number of mice with metastases between two groups. Exact Fisher *t*-test was used to test the difference in metastatic foci numbers among the four groups of mice. Linear correlation analyses were performed to determine the correlation between the gene expression levels. *P* < 0.05 was considered statistically significant.

## Supplementary information

Supplementary Figure 1

Supplementary Figure 2

Supplementary Figure 3

Supplementary Figure legends
