# Supplementary figures and images for: Targeting androgen receptor (AR) with antiandrogen Enzalutamide increases prostate cancer cell invasion yet decreases bladder cancer cell invasion via differentially altering the AR/circRNA-ARC1/miR-125b-2-3p or miR-4736/PPARγ/MMP-9 signals
Source: Cell Death Differ. 2021 Jun 14;28(7):2145–59. doi: 10.1038/s41418-021-00743-w (PMC8257744; doi:10.1038/s41418-021-00743-w)

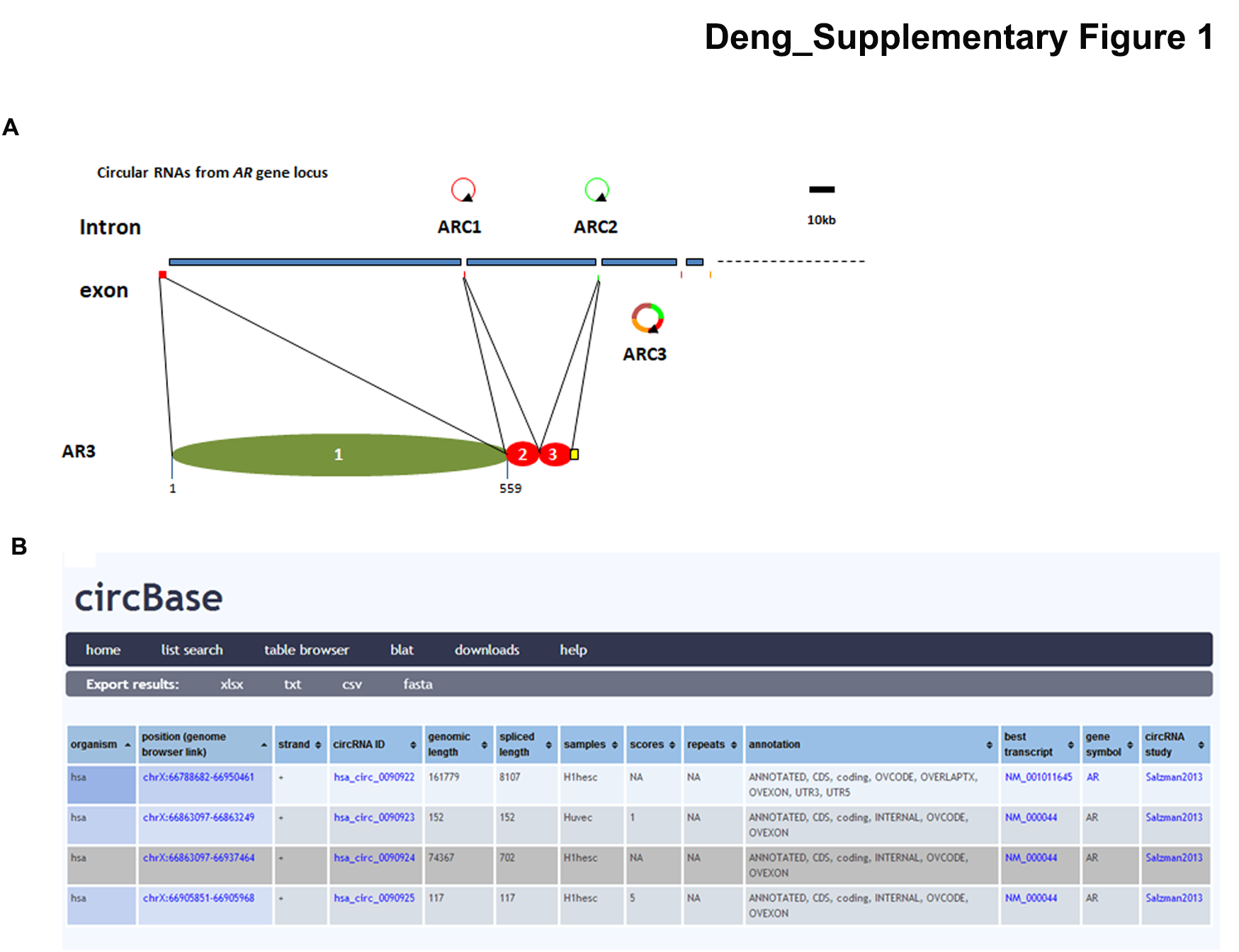

Supplement: Supplementary file 1 — Supplementary Figure 1 [file 41418_2021_743_MOESM1_ESM.tif]

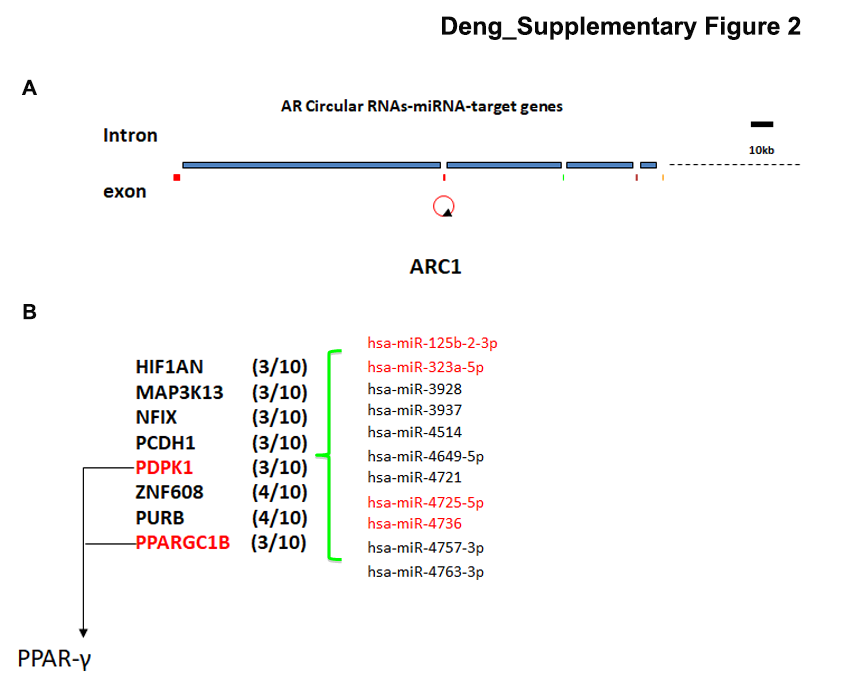

Supplement: Supplementary file 2 — Supplementary Figure 2 [file 41418_2021_743_MOESM2_ESM.tif]

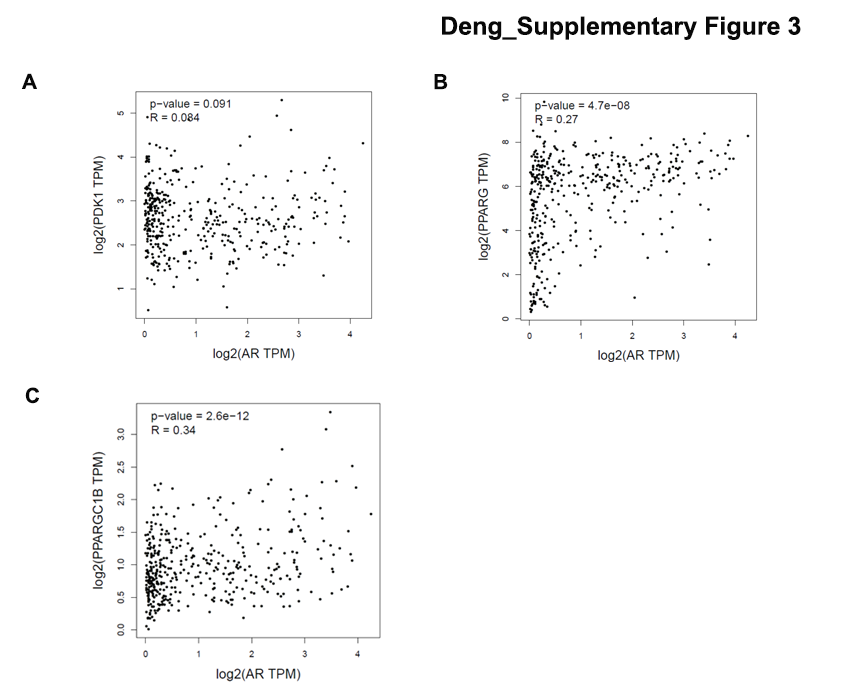

Supplement: Supplementary file 3 — Supplementary Figure 3 [file 41418_2021_743_MOESM3_ESM.tif]
