# Supplementary material for: Targeting androgen receptor (AR) with antiandrogen Enzalutamide increases prostate cancer cell invasion yet decreases bladder cancer cell invasion via differentially altering the AR/circRNA-ARC1/miR-125b-2-3p or miR-4736/PPARγ/MMP-9 signals
Source: Cell Death Differ. 2021 Jun 14;28(7):2145–59. doi: 10.1038/s41418-021-00743-w (PMC8257744; doi:10.1038/s41418-021-00743-w)
Supplement: Supplementary file 4 — Supplementary Figure legends [file 41418_2021_743_MOESM4_ESM.docx]

**Supplementary Figure Legends**

**sFig. 1A.** Bioinformatic prediction and analysis of candidate circRNAs from *AR* gene locus. **B.** The circRNAs from *AR* gene locus in circBase.

**sFig. 2A.** Location and properties of circRNA-ARC1 in *AR* gene locus. **B.** Bioinformatics prediction, literatures search and analysis candidates of AR circRNA-ARC1-related miRNAs target genes.

**sFig. 3A.** The correlation of AR with PDK1 derived from TCGA datasets. **B.** The correlation of AR with PPARγ derived from TCGA datasets. **C.** The correlation of AR with PPARGC1B derived from TCGA datasets.
